# Promoting a structural view of biology for varied audiences: an overview of RCSB PDB resources and experiences

**DOI:** 10.1107/S002188981002371X

**Published:** 2010-08-03

**Authors:** Shuchismita Dutta, Christine Zardecki, David S. Goodsell, Helen M. Berman

**Affiliations:** a RCSB PDB, Rutgers, State University of New Jersey, Newark, New Jersey, USA; b RCSB PDB, Scripps Research Institute, La Jolla, California, USA

**Keywords:** Protein Data Bank, crystallographic education, macromolecular structures, biological crystallography

## Abstract

The Research Collaboratory for Structural Bioinformatics Protein Data Bank (RCSB PDB) supports scientific research and education worldwide by providing an essential resource of information on biomolecular structures. In addition to serving as a deposition, data-processing and distribution center for PDB data, the RCSB PDB offers resources and online materials that different audiences can use to customize their structural biology instruction. These include resources for general audiences that present macromolecular structure in the context of a biological theme, method-based materials for researchers who take a more traditional approach to the presentation of structural science, and materials that mix theme-based and method-based approaches for educators and students. Through these efforts the RCSB PDB aims to enable optimal use of structural data by researchers, educators and students designing and understanding experiments in biology, chemistry and medicine, and by general users making informed decisions about their life and health.

## Abstract 2

The Research Collaboratory for Structural Bioinformatics Protein Data Bank (RCSB PDB) serves a community of users with diverse backgrounds and interests. In addition to processing, archiving and distributing structural data, it also develops educational resources and materials to enable people to utilize PDB data and to further a structural view of biology.

## 1. Introduction

The Protein Data Bank (PDB) is the single data archive for experimentally determined three-dimensional structures of proteins, nucleic acids and their complex assemblies. It was originally established in 1971 at Brookhaven National Laboratories (Bernstein *et al.*, 1977 ▶) with just seven structures. Now, at the time of writing, the PDB archives the coordinates and related experimental data for more than 65 000 structures, mainly determined by X-ray crystallography, nuclear magnetic resonance (NMR) and electron microscopy (EM), with a small number determined by fibre diffraction, neutron diffraction and solution scattering. The archive is managed by the Worldwide Protein Data Bank (wwPDB; Berman *et al.*, 2003 ▶), a group of organizations that act as deposition, data-processing and distribution centers for PDB data.

In the early years, the PDB was used primarily by X-ray crystallographers to deposit and access their structure entries as necessary. As more structures became available, resulting from both advancements in experimental techniques and the requirement by journals and funding agencies to make structural data available, the depositor and user bases expanded. The possibilities afforded by the World Wide Web and new database technologies simplified access and allowed the PDB to become a versatile data resource. Currently, computational biologists require structural data for drug design, structure prediction and other molecular modeling projects. Bio­chemists and molecular biologists use structural data to inform their research, design new experiments and develop new hypotheses. Software developers create visualization and data analysis tools, and database managers create resources for structural bioinformatics. Students, educators and educational-content developers access structures from the PDB to obtain a first-hand view of biological molecules, while general audiences learn about the PDB as news media highlight the role of molecular structures in identifying new discoveries, diagnostics and drugs that affect people’s lives.

When the Research Collaboratory for Structural Bioinformatics (RCSB) PDB was established (Berman *et al.*, 2000 ▶), it was recognized that support should be provided to all users who were already using PDB structures in their research and teaching, and that resources should be made available to introduce new students, teachers and other users to the archive. This article describes the resources and teaching experiences of the RCSB PDB, with a focus on teaching crystallography and the use of structural data for audiences with varied backgrounds and interests.

## 2. Resources for teaching and learning crystallography

When the first protein crystal structures were solved (Perutz *et al.*, 1960 ▶; Kendrew *et al.*, 1958 ▶), teaching and learning X-ray crystallography involved clear comprehension of the physical and mathematical concepts required at all stages of data collection and structure determination. At that time, all depositors and users of PDB data had a clear understanding of structural data and its implications. However, these days many of the steps in determining structures by X-ray crystallography are automated. Moreover, a large group of computational and experimental biologists use three-dimensional structural data from the PDB for their research. Although these audiences are conversant with biomolecular structures, many do not understand the details and assumptions made in determining these structures and how some of the structural details can be identified using PDB data. Lack of information or misconceptions among these audiences have led to some awkward research results and analyses. Method-based resources and instruction provided by the RCSB PDB can assist audiences without expertise in crystallography to use the data optimally.

### 2.1. *Looking at Structures*: an online resource for computational and experimental biologists

The PDB began as an archive for crystallographic data, and about 86% of the current PDB entries are crystal structures. For users without a strong background in crystallography, using PDB data can be very challenging. A series of explanatory pages under the *Looking at Structures* feature of the RCSB PDB website (http://www.pdb.org) guides users through a few of the technical details of PDB structure entries (Goodsell, 2009 ▶). This resource is a response to repeated user questions that relate to an underlying misunderstanding of the information held in PDB entries. It helps users to understand and compare structures of the same or related molecules in the archive.

The best example of how this resource is useful can be seen in understanding the relationship between asymmetric units and biological assemblies. Non-experts are frequently unaware that PDB entries for crystal structures include coordinates for the asymmetric unit (the structurally unique portion of the symmetrical crystal lattice). For oligomeric proteins and complexes, the asymmetric unit may include only part (or occasionally multiple copies) of the biologically relevant assembly of the molecule or complex being studied. Building on work at the European Bioinformatics Institute’s Protein Quaternary Structure (PQS; Henrick & Thornton, 1998 ▶) and Protein Interfaces, Surfaces and Assemblies (PISA; Krissinel & Henrick, 2005 ▶) services, the RCSB PDB website offers coordinates and images for the asymmetric unit and its possible biological assemblies. A section of *Looking at Structures* explains this concept, and directs users on how to access and download the biological assembly coordinates and corresponding images.

The *Looking at Structures* resource continues to expand, covering topics that can pose challenges to new users who are not familiar with the technical details of X-ray crystallography, NMR spectroscopy or EM. Currently, there are pages that describe the information stored in PDB coordinate and data files, describe the structure determination methods (X-ray crystallography, NMR and EM), provide examples of why coordinates of some atoms or residues may be missing in PDB files (such as missing loops or flexible side chains), introduce molecular graphics programs, and illustrate the meaning and implication of crystallographic resolution, *R* value and *R*-free.

### 2.2. *Crystallography For Modelers*: a short course for computational biologists

Based on requests from members of the user community, a short course called *Crystallography for Modelers* was held in May 2009 at Rutgers University. The objective of the course was to help participants understand PDB data and avoid mistakes in their research. The course was aimed at pharmaceutical/biophysical modelers looking for a deeper understanding of crystal structures and their derivation, reliability and interpretation, and proper application and use of PDB files. Students from academia and industry attended the two-day course to hear lectures from RCSB PDB members. Practical aspects (with some theory) of calculation, interpretation and use of electron-density maps, refinement approaches, and validation of structural data were discussed, followed by software demonstrations and hands-on sessions organized by industrial participants and sponsors. The format and content of the course were very well received. The RCSB PDB plans to continue this outreach effort at other venues, and to extend it to include resources more suited to educators and developers of educational materials.

## 3. Resources and experiences promoting a structural view of biology

Structures are now routinely used to understand the overall function of biological macromolecules in health and disease, to study detailed steps in biochemical reaction pathways, for drug design, and to customize disease treatments. With this diversity in PDB structural data usage, there is a growing need to balance method-based education, which is designed for technical users who need to understand the methodological details of structural biology, with the needs of non-specialists who are also interested in a molecular structural description of biology. Modern structural biology education needs to be flexible to match the background and needs of these different audiences.

The first step in the creation and promotion of resources that support a structural view of biology was the establishment of the *Molecule of the Month*, a resource for general audiences that takes a theme-based approach to describing the connections between PDB structures and the reader’s own life and health. The RCSB PDB has also developed materials and courses to bring structural data into undergraduate and graduate-level classrooms. These resources mix theme-based and method-based approaches, providing information on the biology behind the structures, but also training students and educators in the use of the PDB and its tools.

### 3.1. *Molecule of the Month*: an online resource for general audiences

The *Molecule of the Month* is designed to encourage new non-expert users to explore and utilize the PDB archive. Since general audiences are more likely to relate to lessons describing particular molecules rather than a discussion of X-ray crystallographic methods, each month a new feature is published on the RCSB PDB home page that highlights the structure and function of a specific molecule or complex, and illustrates how it relates to topics in human health, welfare and technology.

The features are carefully crafted to appeal to general audiences, with text that avoids jargon and colorful graphic molecular imagery. A non-photorealistic space-filling representation is used for most structures to provide an easily comprehended image of the shape and form of the molecule (Goodsell, 2005 ▶). Most of the static images support novice users, but interactive views are also available. The features are available as web pages and in PDF format. High-resolution files of the *Molecule of the Month* images are also available for download.

**Figure 1:**
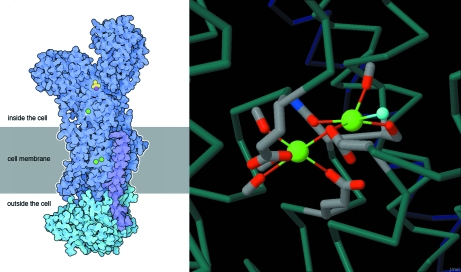
Images from the October 2009 *Molecule of the Month* (doi.org/10.2210/rcsb_pdb/mom_2009_10). Static images such as the illustration on the left are available for download as high-resolution TIFF files; *Jmol* images, as on the right, launch interactive curated views of a structure.

The *Exploring the Structure* section contains links to curated *Jmol* views (http://www.jmol.org/) and links to the RCSB PDB’s *Ligand Explorer* (Moreland *et al.*, 2005 ▶), with viewing suggestions to explore the surroundings of ions, inhibitors or drugs bound in the structure. For example, the *Molecule of the Month* feature on the sodium–potassium pump (Fig. 1 ▶) illustrates a description of the pumping cycle with an interactive *Jmol* view in the middle of the cycle, when the pump has just picked up potassium ions. Instructions are given for using the Java applet *Ligand Explorer* to display the interaction between the two metal ions.

The features also offer *Topics for Further Exploration*, which are thought-provoking questions relating to the structure and function of the molecule described, and *References* for additional reading.

Throughout the text, links are provided to other resources at the RCSB PDB site, including PDB entries and related *Molecule of the Month* features. In this way, these features offer entry points to the more than 65 000 structures in the PDB archive. A search for lysozyme will return more than 1000 PDB entries that represent lysozyme structures from different source organisms, with different mutations, levels of resolution, bound ligands and more. The *Molecule of the Month* column on this topic provides carefully selected and annotated lysozyme entries for users to start exploration. The major goal is to provide initial examples to help new users start using the database.

The *Molecule of the Month* features are one of the most visited resources at the RCSB PDB website. A detailed analysis of traffic through the *Molecule of the Month* pages revealed that the archive of past columns (http://www.pdb.org/pdb/motm.do) continues to be extensively accessed, with interesting spikes of activity. For instance, visits to the hemagglutinin and neuraminidase features increased during the H1N1 flu season. This analysis also revealed that many users reach the *Molecule of the Month* through Wikipedia, providing a convenient path from a popular online resource to the RCSB PDB, and that most users continue exploration of the overall website after reaching the *Molecule of the Month*. Based on anecdotal reports, the archive of these features is also widely used by high-school educators in developing lessons, by students in researching their lessons, and by developers of Science Olympiad protein-modeling events in designing questions and exercises.

### 3.2. *Students Exploring Molecular Structures* (SEMS) courses

The RCSB PDB members at Rutgers, the State University of New Jersey, have created theme-based structural biology courses for undergraduate curricula. The first of these courses was offered as an honors seminar in 2006, with the human digestive system as the theme. Similar undergraduate honors seminars have been offered with cancer and acquired immune deficiency syndrome (AIDS) as the themes in 2008 and the human nervous system as the theme in 2010. Additionally, summer internships (2006 and 2008), independent study projects (2010) and even graduate courses in biophysical chemistry (2006 and 2008) have been offered at Rutgers using this course format.

In all of these courses, students were introduced to fundamental concepts in protein and nucleic acid structure, and to experimental methods used for structure determination (X-ray crystallography, NMR and EM). Students learned to use the RCSB PDB and its related tools and resources for structure visualization and analysis. After this initial preparation, students were introduced to the specific course theme by an expert in the field. Students were then assigned molecules related to the theme for their structure explorations. They identified the structures of these molecules in the PDB, read the primary citations and related articles, and visualized and analyzed relevant structures to understand structure–function and disease associations of the molecule. Students made oral presentations and wrote online reports with original images and textual descriptions of a molecular structural view of the theme being studied. Through this process, students learned a structural view of the course theme, and were also exposed to scholarly research, reading and writing scientific papers, and presentation of their analysis. The online reports were submitted using a framework based on a content management system, allowing students access to their reports (text and images) online and the flexibility to publish the reports only when complete.

Assessment of student performance in class, student and faculty feedback about the course, and student self-assessment surveys (such as SALG, http://www.salgsite.org/) all suggest that the theme-based first-hand experience of exploring actual protein structures inspired students to learn about structural biology. Students gained a new perspective on the subject and learned skills that they could apply to other courses in the curriculum.

**Figure 2:**
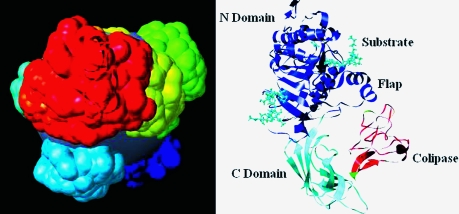
Assessing the success of the undergraduate honors seminar class: images of biological molecules generated by a student. The left image shows a structure with multiple models generated by the student at the beginning of the class. By the end of the class the student was able to create clear figures, shown on the right, with appropriate regions of the structure drawn and labeled distinctly.

The success of these courses can also be measured by following a student’s ability to create images of proteins from the beginning to the end of the class (Fig. 2 ▶). By the end of each semester, the majority of the students were able to read, comprehend and report clearly on their assigned theme-related molecules. Students with no background in biology (non-science majors) also excelled in these courses. In 2006 and 2008, many students returned for summer internships at the RCSB PDB, while some students joined structural biology laboratories at Rutgers and other institutions, where they are involved in research.

Multiple course offerings using the SEMS format have provided an opportunity to examine variations related to the choice of topics, the instructor, and the students’ background knowledge and interests. In the next few years, this course format will be tested at a variety of colleges and universities in a number of courses and disciplines in biology and chemistry. The hope is to assess the possibility of distributing a transferable module and online framework that can be combined with a specific theme or topic and offered as a new SEMS course at institutions around the US. This course format has the potential to provide any undergraduate teaching faculty with the ability to incorporate a structural perspective in their course design. For faculties with limited or no background in X-ray crystallography or other experimental structural determination methods, the transferable module will provide material to introduce fundamental concepts, tools and resources related to structural biology. The online student-reporting framework will allow the teaching faculty to collaborate with experts for help with evaluation of the students’ reports.

### 3.3. *Molecular Anatomy Project*: an online educational resource in the making

In order to describe the workings of an entire organism in structural terms, the *Molecular Anatomy Project* (MAP) was initiated as part of a summer internship program in 2005. The goal of this ambitious project is to create a web resource that provides a structural view of all human molecules and describes molecular structural perturbations and their impact on health and disease. In this resource, structural descriptions of all human molecules are organized by organ system (digestive system, immune system, nervous system *etc.*), organ and tissue type (stomach, liver, pancreas *etc.*), and disease associations. The project is built on the same online framework as is used for the SEMS courses, making it easily manageable. In fact, the MAP project was the initial motivation for creating the SEMS courses so that the images and descriptions from the student reports could be included in the MAP resource.

Over the course of many years, the MAP resource is being populated by undergraduate and graduate student reports at Rutgers. Since many researchers, students and educators rely on the information presented by the RCSB PDB, the student-authored content will be made publicly available to all users after a period of critical review. This will ensure that MAP can act as a reliable gateway to the PDB archive for exploration, analysis and comparison of macromolecular structures, for example within or between organ systems, within or between different organs and tissues, and in various disease pathways.

## 4. Resources for education

Knowledge of biomolecular structure is essential for all fields of biology, and an increasingly structural approach is being used in biology classrooms. The RCSB PDB resources for education provide ideas, materials and training to allow students and educators to develop an interest in molecular structures, find information that is relevant to the subjects they are studying and access PDB data.

### 4.1. The RCSB PDB educational resources

The *Educational Resources* section (Table 1 ▶) provides worldwide distribution of RCSB PDB-related tools, posters, tutorials, activities and lesson plans. Many of these resources are aimed at middle and secondary education, and are presented and distributed at professional society meetings. For example, the New Jersey Science Convention, co-sponsored by the New Jersey Science Teachers Association and the New Jersey Science Education Leadership Association (http://www.njsc-online.com/), has served as a test-bed for a number of the educational materials, activities and tutorials that are now available from *Educational Resources*. Examples of how educators use PDB data and RCSB PDB resources in their teaching are published quarterly, in the *Education Corner* feature of the RCSB PDB newsletter. The goal is to provide enough background and materials to make science teachers interested in incorporating biological structure into their curricula and lesson plans.

The RCSB PDB website is supported by help pages and a help desk to assist users at all levels.  Comprehensive training materials to help beginners utilize RCSB PDB features and functionality are also freely available at http://www.openhelix.com.  Developed in collaboration with the RCSB PDB, the training tools include an online narrated tutorial that demonstrates basic and advanced searches, how to generate reports, the different options for exploring individual structures, and using many of the resources and tools available at the RCSB PDB for research and education. The full tutorial runs for about an hour and can be navigated by specific chapters. Animated presentation slides of the tutorial, slide handouts and exercises are available for download and for teachers and professors to create classroom content.

### 4.2. Outreach efforts

RCSB PDB staff members hold courses, workshops and demonstrations to assist undergraduate lecturers in the use of RCSB PDB resources for teaching in their classrooms. RCSB PDB members also teach various courses and interact with students at their host universities (Rutgers in New Jersey and University of California at San Diego) to promote a structural view of biology.

The RCSB PDB sponsors tours from local high schools and provides an internationally travelling art exhibit of images from the RCSB PDB. It also sponsors the protein-modeling trial event (http://education.pdb.org/olympiad) at the New Jersey Science Olympiad competitions. In this national level event, as designed by the Center for Biomolecular Modeling at the Milwaukee School of Engineering (http://cbm.msoe.edu/), student teams demonstrate their understanding of protein structure and function through hand-built three-dimensional models of specific proteins. Since 2006, New Jersey schools have had the unique opportunity of having their models judged by the RCSB PDB annotation staff. All these experiences with teachers and students often lead to new initiatives and collaborations.

Other outreach efforts, aimed at introducing students to the world of biological macromolecules, include large-scale science fairs and expositions, where an interest is developed by making links between three-dimensional structures in health and in disease, and by allowing students to visualize some of the structural concepts that they read about in journals and text books. Viruses, for example, can be built using a PDF template, available from the RCSB PDB website, which can be folded into an icosahedral shape. This activity has been used at many events to introduce students to concepts in symmetry and molecular structure, and to an understanding of diseases.

The success of all educational efforts is evaluated through pre- and post-content knowledge surveys, opinion surveys, feedback collected from professional society meetings and electronic help desks, and from an increasing demand for resources from teachers and students.

## 5. Future

Educational programs and resources (both method-based and theme-based) will continue to be developed to help researchers, teachers, students and general users at all levels to understand PDB data and structures. Feedback from these efforts is always used to develop the resources further. The RCSB PDB intends to use current resources and staffing to the full in order to promote a better understanding and utilization of biomolecular structures by audiences around the world.

## Figures and Tables

**Table 1:** Educational resources from the RCSB PDB website In addition to the *Looking at Structures* and *Molecule of the Month* features, the RCSB PDB offers a variety of downloadable tools and resources.

*Molecules in Motion Kiosk Viewer*	A full-screen animation program that displays structures rotating at different angles and from different perspectives, and focuses on chemical components within the structure
	
Posters	*Molecular Machinery: A Tour of the Protein Data Bank* features illustrations of 75 selected structures, shows their relative sizes at a scale of three million to one and generally describes their critical roles in the functions of living cells
	*How Do Drugs Work?* uses examples from the PDB archive to illustrate different types of drugs
	
Calendars	These calenders feature stylized illustrations and detailed structural descriptions
	
Website tutorials	These Flash tutorials developed by RCSB PDB demonstrate how to use the RCSB PDB tools Advanced Search and the Medical Subject Headings (MeSH) browser (Sayers *et al.*, 2010 ▶), and the molecular viewers *Protein Workshop*, *KiNG* (Chen *et al.*, 2009 ▶) and *Jmol*
	Free narrated tutorial and supplementary materials, developed in collaboration with Openhelix, demonstrate how to use the site
	
Animations	Animations of *Molecule of the Month* images for the ribosome and for hemoglobin are available for download
	An interview describing how to use PDB data in animation is also available
	
Activities and lessons	*Exploring the Bioinformatics of Green Fluorescent Protein* examines a GFP protein at different resources
	*Handheld 3D Virus Model* can be used to make a paper model of the dengue virus
	A lecture and lesson plans describing *Virus Shape and Structure* and the *Life Cycle of the Dengue Virus* are available for middle- and high-school classrooms
	
Exhibit materials	The *Art of Science* traveling exhibit includes pictures and captions from the RCSB PDB†
	The *Sea of Genes* Flash animation about specific proteins found in marine organisms is used in an exhibit at the Birch Aquarium (La Jolla, California)
	
RCSB PDB poster prize	Awarded for the best student poster presentation at a number of different meetings

† If you would be interested in sponsoring this exhibit at your institution or would like more information, please contact us at info@rcsb.org.

## References

[bb1] Berman, H. M., Henrick, K. & Nakamura, H. (2003). *Nat. Struct. Biol.* **10**, 980. 10.1038/nsb1203-980 14634627

[bb2] Berman, H. M., Westbrook, J., Feng, Z., Gilliland, G., Bhat, T. N., Weissig, H., Shindyalov, I. N. & Bourne, P. E. (2000). *Nucleic Acids Res.* **28**, 235–242. 10.1093/nar/28.1.235 PMC102472 10592235

[bb3] Bernstein, F. C., Koetzle, T. F., Williams, G. J. B., Meyer, E. F. Jr, Brice, M. D., Rodgers, J. R., Kennard, O., Shimanouchi, T. & Tasumi, M. (1977).* J. Mol. Biol.* **112**, 535–542. 10.1016/s0022-2836(77)80200-3 875032

[bb4] Chen, V. B., Davis, I. W. & Richardson, D. C. (2009).* Protein Sci.* **18**, 2403–2409. 10.1002/pro.250 PMC2788294 19768809

[bb5] Goodsell, D. S. (2005).* Structure*, **13**, 347–354. 10.1016/j.str.2005.01.012 15766535

[bb6] Goodsell, D. S. (2009).* J. Biocommun.* **35**, E52–E57.

[bb7] Henrick, K. & Thornton, J. M. (1998).* Trends Biochem. Sci.* **23**, 358–361. 10.1016/s0968-0004(98)01253-5 9787643

[bb9] Kendrew, J. C., Bodo, G., Dintzis, H. M., Parrish, R. G., Wyckoff, H. & Phillips, D. C. (1958).* Nature* (*London*), **181**, 662–666. 10.1038/181662a0 13517261

[bb10] Krissinel, E. & Henrick, K. (2005). *Computational Life Sciences* First International Symposium (CompLife 2005), Konstanz, Germany, September 25–27, 2005. *Proceedings*, edited by M. R. Berthold, R. Glen, K. Diederichs, O. Kohlbacher & I. Fischer, pp. 163–174. Berlin: Springer-Verlag.

[bb11] Moreland, J. L., Gramada, A., Buzko, O. V., Zhang, Q. & Bourne, P. E. (2005).* BMC Bioinformatics*, **6**, 21. 10.1186/1471-2105-6-21 PMC548701 15694009

[bb12] Perutz, M. F., Rossmann, M. G., Cullis, A. F., Muirhead, G. & Will, G. (1960).* Nature* (*London*), **185**, 416–422. 10.1038/185416a0 18990801

[bb13] Sayers, E. W. *et al.* (2010). *Nucleic Acids Res.* **38**, D5–16. 10.1093/nar/gkp967 PMC2808881 19910364

